# Oncological Outcomes of Partial Gland Ablation Using High-Intensity Focused Ultrasound After Additional Confirmatory Transperineal Mapping Biopsy in Men with Prostate Cancer

**DOI:** 10.3390/biomedicines12112487

**Published:** 2024-10-30

**Authors:** Jihwan Lee, Wan Song

**Affiliations:** Department of Urology, Samsung Medical Center, Sungkyunkwan University School of Medicine, Seoul 06351, Republic of Korea; aoke12@gmail.com

**Keywords:** prostate cancer, partial gland ablation, HIFU, perineal biopsy, recurrence

## Abstract

Background/Objectives: To evaluate whether additional confirmatory transperineal mapping biopsy (TPMB) in men with localized prostate cancer (PCa) alters the treatment plan and outcome of partial gland ablation (PGA) using high-intensity focused ultrasound (HIFU). Methods: We retrospectively reviewed data from 96 patients who underwent PGA using HIFU between January 2020 and June 2022. After multiparametric magnetic resonance imaging (mpMRI), all men underwent transrectal ultrasound (TRUS)-guided, cognitive-targeted biopsy and systematic biopsy. Men eligible for PGA using HIFU first underwent confirmatory TPMB. Any changes in the treatment plan after TPMB were analyzed. Follow-up TRUS-guided biopsy was performed 1 year post-operatively to evaluate oncological outcomes. Clinically significant PCa (csPCa) was defined as Gleason grade (GG) ≥ 2. Results: Among all subjects, the median age (IQR) was 65.0 (60.0–72.0) years and the prostate-specific antigen level was 5.20 (3.71–7.81) ng/mL. The results of both TRUS-guided biopsy and TPMB led to a change in the treatment plan (from unilateral to bilateral PGA) for 13 (13.5%) patients. The 1-year follow-up TRUS-guided biopsy identified PCa in 13 (13.5%) patients, and csPCa in 7 (7.3%) patients. The infield- and outfield-positive rates were 8.3% (8/96) and 3.1% (3/96), respectively, for any PCa, and 3.1% (3/96) and 2.1% (2/96), respectively, for csPCa. Conclusions: Confirmatory TPMB results in better disease identification and localization, thereby affecting the treatment plan and improving oncological outcomes. Therefore, confirmatory TPMB should be considered to establish an appropriate strategy for patients with localized PCa eligible for PGA using HIFU.

## 1. Introduction

According to the cancer statistics in 2020, prostate cancer (PCa) was the cancer type with the second highest incidence and the fifth highest mortality rate in men worldwide [1]. The standard treatment for localized PCa is to treat the entire prostate (i.e., radical prostatectomy (RP) or radiation therapy (RT)) [2,3]; however, complications that reduce quality of life are common [4,5,6,7]. In selected men with a very low or low risk of PCa, active surveillance (AS) is regarded as a viable treatment option. However, this can raise concerns about progression, and repeated imaging and biopsies cause discomfort [8]. Therefore, there have been many efforts to improve oncological outcomes while maintaining functional outcomes. With this in mind, high-intensity focused ultrasound (HIFU) may act as a bridge between AS and RP or RT [9].

HIFU is a method of tissue ablation achieved by delivering ultrasound waves that are converted to heat without damaging adjacent structures such as neurovascular bundles, seminal vesicles, and the prostatic capsule. However, as there is a lack of long-term and randomized data, the European Association of Urology (EAU) guidelines recommended that HIFU should be performed as a part of a clinical trial or well-designed prospective study in selected patients [10,11]. To maximize the outcomes of HIFU, it is important to determine the localization of the PCa. Multiparametric magnetic resonance imaging (mpMRI) interpreted by the Prostate Imaging Reporting and Data System (PI-RADS) is used widely as an essential diagnostic tool for in men with suspected PCa [12]. The gold standard for PCa detection/confirmation is transrectal ultrasound (TRUS)-guided biopsy [13,14]; however, PCa is multifocal in most patients, and a significant proportion of PCa cases may be missed, despite improvements in PCa detection rates using contemporary imaging modalities [15,16].

Transperineal mapping biopsy (TPMB) is an alternative method of prostate biopsy that has the advantage of being able to assess the entire prostate, including the anterior section [17]. Thus, TPMB can improve detection of PCa by about 30–45% when compared with TRUS-guided biopsy; in addition, it can confirm the location of the cancer accurately and in three dimensions [18,19]. However, few studies have examined the effects of TPMB on the performance of partial gland ablation (PGA) with HIFU. Therefore, we reported our early clinical experience of PGA with HIFU and evaluated whether additional confirmatory TPMB alters the treatment plan and increases the oncological outcomes of PGA with HIFU in men with localized PCa.

## 2. Materials and Methods

### 2.1. Study Design

This study was approved by the Institutional Review Board of the Samsung Medical Center (IRB No: 2018-01-082). The requirement for informed consent was waived due to the retrospective nature of the study design. All study protocols were performed in accordance with the Declaration of Helsinki, and patient data complied with the relevant data protection and privacy regulations.

The medical charts of 107 patients who underwent PGA using HIFU for localized PCa between January 2020 and June 2022 were reviewed retrospectively. The inclusion criteria were as follows: (1) serum prostate-specific antigen (PSA) levels < 10 ng/mL; (2) unilateral disease based on TRUS-guided biopsy and mpMRI imaging; (3) number of positive cores ≤ 3; (4) Gleason grade (GG) ≤ 3; (5) a distance of more than 5 mm from the apex to the index lesion; and (6) participants underwent confirmatory TPMB. The exclusion criteria were as follows: (1) previous transurethral resection of the prostate (TURP); (2) urinary tract infection (UTI) or acute bacterial prostatitis; (3) severe prostatic calcification; and (4) a narrow anus that prevented insertion of a HIFU probe under general anesthesia. Of the entire cohort, 11 men were excluded due to incomplete clinical data; therefore, 96 men were included in the final analysis.

### 2.2. Data Collection

Demographic and pathologic data, including age, body mass index, history of hypertension and diabetes mellitus, PSA level, and prostate volume, were collected from medical charts. Prostate volume was assessed by TRUS and calculated using the ellipsoid formula: width × length × height × 0.52. PSA density was calculated by dividing the total PSA by the prostate volume. The number of total, targeted, and positive cores, as well as the maximum percentage tumor involvement in a positive core, were also identified.

### 2.3. mpMRI Protocol and Data Interpretation

mpMRI was performed using a 3.0-tesla MRI instrument (Intera Achieva TX, Philips Healthcare, Best, The Netherlands) with a 6-channel, phase-array body coil. The protocol was as follows: T1-weighted, T2-weighted, and diffusion-weighted imaging with b values of 0, 100, and 1000 s/mm^2^, and dynamic contrast-enhanced imaging. All images were acquired in accordance with ESUR guidelines [20].

A picture archiving and communication system (Centricity, GE Healthcare, Barrington, IL, USA) was used to load all images, and they were interpreted by three uro-radiologists with expertise in prostate imaging. The index lesion was scored according to the PI-RADS v2 using a 5-point scale [17].

### 2.4. Protocol for TRUS-Guided Biopsy

Biopsy was preceded by rectal preparation using a bisacodyl suppository. Oral quinolone and/or third generation cephalosporin as prophylactic antibiotics were taken for 1 week, starting on the day before the procedure. First, a cognitive-targeted biopsy of more than one core was performed for the suspicious lesion; this was followed by a 12-core systematic biopsy using an 18-gauge core biopsy needle mounted to an automatic biopsy gun in a 2-dimensional plane (axial and sagittal) TRUS probe (BK Medical, Herlev, Denmark; Transducer 8818). All biopsies were performed as outpatient procedures under local anesthesia.

### 2.5. Protocol for TPMB

Men eligible for PGA using HIFU underwent confirmatory TPMB. Preparation for the procedure was the same as for TRUS-guided biopsy, although aminoglycoside (amikacin 500 mg) was also administered intramuscularly a few minutes before biopsy.

TPMB was performed under general anesthesia to ensure that the patient did not move during the procedure. Patients were repositioned in an extended lithotomy position. At first, a digital rectal examination was performed, followed by TPMB using a dual-plane TRUS 8848 brachytherapy probe (BK Medical, Herlev, Denmark) capped with a water-filled brachytherapy balloon and a Classic STEPPERTM device (CIVCO, Kalona, IA, USA). Biopsy was performed using an 18 G, 20 cm-long biopsy needle (ACECUT; TSK Laboratory, Tochigi, Japan) through a standard brachytherapy grid at 5 mm intervals; the whole prostate gland was divided into anterior, mid, and posterior regions on the left and right side, and biopsies were performed through the perineum on the medial and lateral sides, respectively [21,22]. The number of longitudinal biopsies taken was dependent on the distance from the apex to the base of the prostate (based on 2 cm); thus, the number of cores was determined ultimately by the size of the prostate. All patients were hospitalized after the procedure and discharged after 1 day of observation.

### 2.6. Histopathologic Analysis

Histology of the biopsy specimens was reported according to the 2014 International Society of Urological Pathology (ISUP) consensus conference guidelines [23]. In the present study, clinically significant PCa (csPCa) was defined as GG ≥ 2.

### 2.7. Protocol for HIFU

A 20Fr, three-way Foley catheter was inserted into the urethra under general anesthesia. The patient was placed in the right decubitus position with the hip joint flexed at 90° and fixed on the operating table. During patient positioning, ultrasound and mpMRI images were fused, and then a HIFU probe was inserted into the anus. The fused images were used to identify the location of the target lesion; PGA was performed using the Focal One^®^ device (Edaps TMS, Vaulx-en-Velin, France). The distal 5 mm of the prostate apex and the proximal 5 mm of the prostate base were preserved to prevent urinary incontinence and contracture of the bladder neck. Patients were discharged 2 days after removing the Foley catheter and self-voiding was confirmed.

### 2.8. Follow-Up Protocol

The patients were followed up according to institutional protocols. In general, they returned 1 month post-operation to undergo a physical examination and check voiding status. After that, they were followed up every 6 months to check PSA levels and voiding status. A prostate mpMRI is recommended annually to check the suspicious lesion. A post-procedure biopsy following the protocol of TRUS-guided biopsy is recommended routinely after 1 year, regardless of serum PSA levels and mpMRI results. The results of biopsy after PGA were categorized as infield, outfield, or both. Infield-positive was defined as the recurrence of tumor within the area that was directly treated by PGA using HIFU. Outfield-positive was defined as the recurrence of tumor outside the area that was treated by PGA using HIFU.

### 2.9. Statistical Analysis

Quantitative variables are presented as the median (interquartile range, IQR) and mean (standard deviation, SD), and qualitative variables as absolute values (percentages). Descriptive statistics were performed for demographic variables. All statistical analyses were performed using IBM SPSS for Windows, version 23.0 (IBM Corp., Armonk, NY, USA).

## 3. Results

The clinical characteristics of the 96 study subjects are summarized in Table 1. The median (IQR) age was 65.0 (60.0–72.0) years, and the median PSA and prostate volume were 5.20 (3.71–7.81) ng/mL and 34.6 (25.5–46.1) mL, respectively. Regarding mpMRI, the PI-RADS scores were as follows: PI-RADS score 1–2 for 25 (26.0) patients, 3 for 57 (59.4) patients, and 4 for 14 (14.6%) patients.

The results of TRUS-guided biopsy and TPMB are summarized and compared in Table 2. The median number of total and positive cores on TRUS-guided biopsy was 12.0 (12.0–12.0) and 1.0 (1.0–2.0), respectively. The GG after TRUS-guided biopsy was as follows: GG1 for 86 (89.6%) patients, GG2 for 8 (8.3%) patients, and GG3 for 2 (2.1%) patients. For TPMB, the median number of total and positive cores was 26 (24.0–36.0) and 2.0 (1.0–3.0), respectively, and GG2 and GG3 was identified in nine (9.4%) and two (2.1%) patients, respectively. Benign prostatic hyperplasia was confirmed in 25 (26.0) patients.

**Figure 1:**
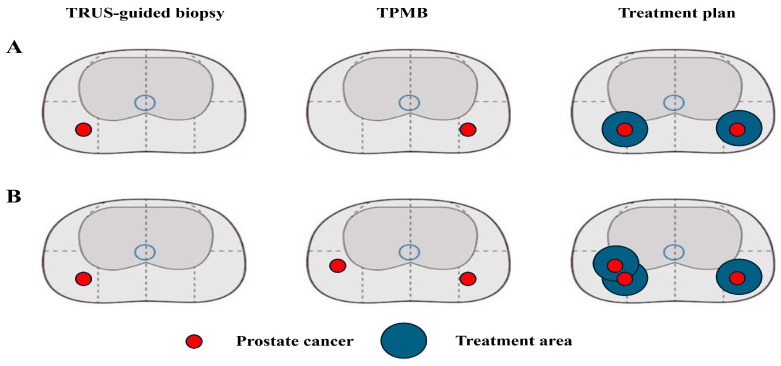
Change of treatment plan after confirmatory transperineal mapping biopsy (TPMB). (**A**) PCa with GG1 was identified on the right side in TRUS-guided biopsy. TPMB biopsy revealed another PCa with GG1 on the left side; thus, the treatment plan changed from right to bilateral partial gland ablation (PGA). (**B**) PCa with GG1 was identified on the right side in TRUS-guided biopsy. TPMB identified not only nearby PCa with GG1 on the right side but also another PCa with GG1 on the left side; thus, the treatment area was expanded in the right side and the treatment plan changed from right to bilateral PGA.

When the results of both TRUS-guided biopsy and TPMB were considered, the treatment plan for 13 (13.5%) patients was changed. Table 3 summarizes the biopsy results and changes in the treatment plan. In 6/13 patients, PCa was identified unilaterally in TRUS-guided biopsy. However, TPMB confirmed PCa on the side opposite to that identified by TRUS-guided biopsy. GG1 and PCa of 1–3 positive cores were located locally; thus, the treatment plan changed from unilateral PGA to bilateral PGA (Figure 1A, N1,2,3,5,6, and 11). For the remaining seven patients, PCa was confirmed not only in the area identified by TRUS-guided biopsy, but also on the opposite side. PCa was identified as almost identical to the location found in the TRUS-guided biopsy, and PCa on the opposite side was also a candidate for PGA; therefore, the treatment plan changed from unilateral PGA to bilateral PGA (Figure 1B, N4,7,8,9,10,12, and 13).

The results of the 1-year follow-up are presented in Table 4. Of the 96 patients, any PCa was identified in 13 (13.5%), and 7 (7.3%) had csPCa. Considering the treatment area, the chance of an infield-positive rate was 8.3% (8/96) for any PCa, but only 3.1% (3/96) for csPCa. The chance of an outfield-positive rate was 3.1% (3/96) for any PCa, and 2.1% (2/96) for csPCa.

## 4. Discussion

Here, we investigated whether additional confirmatory TPMB led to a decision to change the treatment plan and/or altered oncological outcomes in men with localized PCa planning to undergo PGA using HIFU. We analyzed 96 patients and found that the treatment plans for 13 (13.5%) patients were changed after confirmatory TPMB. The 1-year follow-up TRUS-guided biopsy revealed that the infield-, outfield-, and both positive rates were 8.3%, 3.1%, and 2.1%, respectively, for any PCa, and 3.1%, 2.1%, and 2.1%, respectively, for csPCa. These results indicate that confirmatory TPMB improves the shortcomings of mpMRI-based TRUS-guided biopsy, thereby helping to improve the oncological results of PGA using HIFU.

mpMRI is an evolving imaging modality that, when used with TRUS-guided biopsy, has improved the PCa detection rate markedly. A recent systematic review reported that among men with PCa confirmed by mpMRI-based targeted biopsy, 72–87% of those with a previously negative biopsy had csPCa [24]. In addition, csPCa detection rates improved by up to 20% when using mpMRI-based targeted biopsy compared with conventional systematic biopsy. Among men with low-risk PCa, who tend to be candidates for AS, a confirmatory biopsy based on mpMRI, coupled with risk stratification, can identify those who are really suitable for AS. These data suggested that mpMRI-based targeted biopsy may improve detection of csPCa, thereby improving clinical risk assessment.

However, TRUS-guided biopsy can miss a significant proportion of csPCa; therefore, TPMB can be a way to compensate for this. A study by Taira AV et al. [18] showed that TPMB accessed the anterior and apical aspect of the prostate easily, with a high rate of PCa detection in these areas of the prostate; therefore, it is recommended for patients under AS, or those undergoing subtotal gland or minimally invasive treatment. In addition, a study by Nafie S et al. [25] reported patients with a previously negative biopsy, but persistent elevation of PSA, who underwent conventional systematic biopsy and 36-core TPMB at the same time. More than half of PCa cases were identified in the anterior area and approximately one-third were identified only by TPMB. These results indicate that TPMB may be superior with respect to disease identification and localization, thereby increasing the chances of implementing an appropriate strategy most suited to the patient’s disease state.

In addition, a study by Hong SK et al. [16] reported the oncological outcomes for 163 patients after PGA with HIFU for clinically unilateral PCa. The inclusion criteria were similar to those of the present study, and only an mpMRI-based targeted biopsy and a 12-core systematic biopsy were performed before PGA. In that study, the 1-year follow-up TRUS-guided biopsy revealed that any PCa was identified in 24.2% of patients, and csPCa was identified in 12.6% of patients, with outfield-positive rates of 9.7% for any PCa and 2.9% for csPCa. In our study, the treatment plan for 13 (13.5%) patients was changed after confirmatory TPMB. The 1-year follow-up TRUS-guided biopsy revealed that PCa was identified in 13.5%, and csPCa in 7.3%, of patients, with outfield-positive rates of 3.1% for any PCa and 2.1% for csPCa. Although direct comparison is difficult, these results indicate that confirmatory TPMB overcomes the shortcomings of mpMRI-based TRUS-guided biopsy, and improves the oncological outcome of PGA using HIFU.

Furthermore, a multi-institutional prospective study reported the oncological outcomes of 111 patients who underwent hemiablation for unilateral PCa [26]. Patients underwent a mpMRI-based targeted biopsy and 12-core systematic biopsy. It is noteworthy that in this study re-biopsy was performed in patients with a suspicious lesion on the contralateral side on mpMRI. Assessment of the follow-up biopsy identified any PCa in 32.7% (33/101) and csPCa in 11.9% (12/101) of patients. Of the 12 patients with csPCa, 7 had lesions on the contralateral side (where hemiablation was not performed). These results suggest that biopsy of suspicious lesions detected by mpMRI is not sufficient, and that a more accurate diagnostic biopsy such as TPMB should be performed to improve oncological outcomes.

Recently, the indications for HIFU were expanded, and studies reporting the use of HIFU as the primary treatment for intermediate-to-high-risk PCa are coming to light. A study by Bass R et al. [27] reported that 88.6% (147/166) of procedures were performed in patients with GG 2-or-greater disease. Although treatment of the whole gland could be avoided in 81% of patients, PCa was not identified in 29.9% of cases, and csPCa was identified in 42% of cases, after TRUS-guided biopsy conducted approximately 1 year post-procedure. Patients with a high risk of recurrence had a higher number of positive cores, or PCa located on the medial side; thus, they received treatment to preserve the urethra. Therefore, TPMB may help to reduce recurrence by predicting multifocality, and by providing an accurate location of the PCa.

Although this analysis has important clinical implications, there are several limitations to be acknowledged. First, this study was conducted retrospectively at a single center, thereby raising concerns about selection bias. In addition, a relatively small number of patients was included, so they likely do not represent all patients eligible for PGA using HIFU. However, we also obtained data prospectively, reflecting actual practice in the real world. Second, we confirmed recurrence using TRUS-guided biopsy after PGA using HIFU, but the results may vary depending on the biopsy method; however, to obtain the best follow-up results, we ensured that the most experienced urologist, who specializes in TRUS-guided biopsy, performed the procedure. Finally, we identified early oncological outcomes after PGA using HIFU, but a longer follow-up is needed to confirm long-term efficacy. Therefore, a large, prospective, randomized study is required to validate the role of TPMB reported here.

## 5. Conclusions

When performing PGA using HIFU for localized PCa, additional use of TPMB helped to establish an optimal treatment plan and improve oncological outcomes. These results indicate that TPMB enables more precise disease identification and localization, thereby increasing the chances of using an appropriate strategy that is suited to the patient’s disease state. Therefore, confirmatory TPMB should be considered in patients with localized PCa eligible for PGA using HIFU.

## Figures and Tables

**Table 1:** Baseline characteristics.

Variables	Value
Number of patients	96 (100)
Age, years	
Median (IQR)	65.0 (60.0–72.0)
Mean (SD)	65.3 (8.7)
Body mass index, kg/m^2^	
Median (IQR)	24.8 (22.8–26.8)
Mean (SD)	24.9 (2.6)
Hypertension, n (%)	48 (50.0)
Diabetes mellitus, n (%)	21 (21.9)
PSA, ng/dL	
Median (IQR)	5.20 (3.71–7.81)
Mean (SD)	6.69 (5.60)
Prostate volume, mL	
Median (IQR)	34.6 (25.5–46.1)
Mean (SD)	38.1 (18.6)
PSA density	
Median (IQR)	0.12 (0.10–0.21)
Mean (SD)	0.16 (0.19)
PI-RADS score in mpMRI	
1–2	25 (26.0)
3	57 (59.4)
4	14 (14.6)
Clinical stage	
cT1	25 (26.0)
cT2a	32 (33.3)
cT2b	22 (22.9)
cT2c	17 (17.8)

IQR, interquartile range; SD, standard deviation; PSA, prostate-specific antigen; PI-RADS, Prostate Imaging Reporting and Data System; mpMRI, multiparametric magnetic resonance imaging.

**Table 2:** Comparison of results of TRUS-guided biopsy with those of transperineal mapping biopsy.

Variables	TRUS-Guided Biopsy	Transperineal Mapping Biopsy
Number of total cores, n (%)		
Median (IQR)	12.0 (12.0–12.0)	26.0 (24.0–36.0)
Mean (SD)	12.2 (1.7)	29.2 (5.8)
Number of positive cores, n (%)		
Median (IQR)	1.0 (1.0–2.0)	2.0 (1.0–3.0)
Mean (SD)	1.8 (1.5)	2.1 (2.1)
Number of target cores, n (%)		
Median (IQR)	2.0 (2.0–3.0)	
Mean (SD)	2.2 (2.1)	
Number of positive target cores, n (%)		
Median (IQR)	1.0 (1–2)	
Mean (SD)	1.5 (1.4)	
Gleason grade, n (%)		
Benign		25 (26.0)
Gleason grade 1	86 (89.6)	60 (62.5)
Gleason grade 2	8 (8.3)	9 (9.4)
Gleason grade 3	2 (2.1)	2 (2.1)
Maximal tumor involvement, %		
Median (IQR)	10 (5.0–25.0)	10.0 (2.5–25.0)
Mean (SD)	17.3 (18.6)	18.5 (24.9)

IQR, interquartile range; SD, standard deviation; TRUS, transrectal ultrasound.

**Table 3:** Changes in the treatment plan after confirmatory transperineal mapping biopsy in men with prostate cancer.

No.	Age	PSA	Prostate Volume	PSAD	TRUS-Guided Biopsy	Transperineal Mapping Biopsy	Treatment Plan	
							From	To
1	45	5.24	28.2	0.19	1/12(+), Left, 3 + 3	1/26(+), Right, 3 + 3	Left PGA	Bilateral PGA
2	61	5.10	35.4	0.14	2/12(+), Right, 3 + 3	1/24(+), Left, 3 + 3	Right PGA	Bilateral PGA
3	63	6.36	33.4	0.19	1/12(+), Right, 3 + 3	3/24(+), Left, 3 + 3	Right PGA	Bilateral PGA
4	72	3.70	45.7	0.08	2/10(+), Right, 3 + 3	3/36, bilateral, 3 + 3	Right PGA	Bilateral PGA
5	70	4.90	35.4	0.14	2/12(+), Right, 3 + 3	1/24(+), Left, 3 + 3	Right PGA	Bilateral PGA
6	61	3.48	20.4	0.17	1/12(+), Right, 3 + 3	2/24(+), Left, 3 + 3	Right PGA	Bilateral PGA
7	55	5.78	61.9	0.09	1/12(+), Right, 3 + 3	4/36(+), bilateral, 3 + 3	Right PGA	Bilateral PGA
8	62	6.42	39.4	0.16	1/12(+), Left, 3 + 3	2/24(+), bilateral,3 + 3	Left PGA	Bilateral PGA
9	71	3.86	44.2	0.09	1/12(+), Right, 3 + 4	2/36, bilateral, 3 + 3	Right PGA	Bilateral PGA
10	59	2.95	26.1	0.11	2/12(+), Right, 3 + 3	2/24(+), bilateral,3 + 3	Right PGA	Bilateral PGA
11	68	4.62	23.0	0.20	1/12(+), Left, 3 + 4	1/24(+), Right, 3 + 3	Left PGA	Bilateral PGA
12	73	7.36	53.6	0.14	1/12(+), Left, 3 + 3	2/36, bilateral, 3 + 3	Left PGA	Bilateral PGA
13	74	8.02	62.5	0.13	1/12(+), Right, 3 + 3	2/36, bilateral, 3 + 3	Right PGA	Bilateral PGA

PSA, prostate-specific antigen; PSAD, prostate-specific antigen density; TRUS, transrectal ultrasound; PGA, partial gland ablation.

**Table 4:** Results of the 1-year follow-up biopsy taken after partial gland ablation using high-intensity focused ultrasound.

	n (%)
Any PCa-positive biopsy after PGA using HIFU	13 (13.5)
Infield-positive	8 (8.3)
Outfield-positive	3 (3.1)
Both positive	2 (2.1)
csPCa-positive biopsy after PGA using HIFU	7 (7.3)
Infield-positive	3 (3.1)
Outfield-positive	2 (2.1)
Both positive	2 (2.1)

PCa, prostate cancer, csPCa, clinically significant prostate cancer; PGA, partial gland ablation; HIFU, high-intensity focused ultrasound.

## Data Availability

The dataset used and/or analyzed during the current study is available from the corresponding author upon reasonable request.
